# Machine Learning-Assisted High-Throughput Molecular Dynamics Simulation of High-Mechanical Performance Carbon Nanotube Structure

**DOI:** 10.3390/nano10122459

**Published:** 2020-12-09

**Authors:** Yi Xiang, Koji Shimoyama, Keiichi Shirasu, Go Yamamoto

**Affiliations:** 1 Department of Aerospace Engineering, Tohoku University, Sendai 980-8579, Japan; xiang.yi.t3@dc.tohoku.ac.jp (Y.X.); keiichi.shirasu.c1@tohoku.ac.jp (K.S.); 2 Institute of Fluid Science, Tohoku University, Sendai 980-8577, Japan; shimoyama@tohoku.ac.jp; 3 School of Mechanical Engineering, Sungkyunkwan University, Suwon 16419, Korea

**Keywords:** carbon nanotube, molecular dynamics simulations, mechanical properties, Frenkel-pair crosslink, machine learning

## Abstract

Carbon nanotubes (CNTs) are novel materials with extraordinary mechanical properties. To gain insight on the design of high-mechanical-performance CNT-reinforced composites, the optimal structure of CNTs with high nominal tensile strength was determined in this study, where the nominal values correspond to the cross-sectional area of the entire specimen, including the hollow core. By using machine learning-assisted high-throughput molecular dynamics (HTMD) simulation, the relationship among the following structural parameters/properties was investigated: diameter, number of walls, chirality, and crosslink density. A database, comprising the various tensile test simulation results, was analyzed using a self-organizing map (SOM). It was observed that the influence of crosslink density on the nominal tensile strength tends to gradually decrease from the outside to the inside; generally, the crosslink density between the outermost wall and its adjacent wall is highly significant. In particular, based on our calculation conditions, five-walled, armchair-type CNTs with an outer diameter of 43.39 Å and crosslink densities (between the inner wall and outer wall) of 1.38 ± 1.16%, 1.13 ± 0.69%, 1.54 ± 0.57%, and 1.36 ± 0.35% were believed to be the optimal structure, with the nominal tensile strength and nominal Young’s modulus reaching approximately 58–64 GPa and 677–698 GPa.

## 1. Introduction

Owing to their excellent mechanical properties, carbon nanotubes (CNTs) have been prominent in several fields since they were first discovered [1,2]. Studies have shown that the Young’s modulus and tensile strength of single-walled CNTs (SWCNTs) are as high as 1 TPa and 100 GPa, respectively [3,4,5]. These mechanical properties make SWCNTs an ideal reinforcing constituent in composites. However, the conventional fabrication techniques adopted in the synthesis processes causes structural disorders in CNTs, which significantly impact their mechanical properties [6,7,8,9]. To overcome this problem, multi-walled CNTs (MWCNTs) that consist of multiple SWCNTs have been considered suitable for practical use [10,11,12,13,14]. Theoretically, if the axial load applied to the MWCNTs is evenly distributed among their walls, these nanotubes can offer an unprecedented load-carrying capacity despite being limited by defects. However, MWCNTs fabricated using the arc-discharge method at high synthesis temperatures often exhibit a type of sword-in-sheath failure [15,16] because the load applied to them is entirely borne by their outermost wall—owing to its high crystallinity—and the van der Waals forces between the adjacent tubes cannot efficiently transfer this load onto their inner walls. On the contrary, employing the traditional chemical vapor deposition method was found to produce fractures in all their walls [16,17]; this is attributed to structural defects arising from low synthesis temperatures that eventually cause the load to be transferred onto the inner walls. Moreover, the nominal tensile strength of these tubes was reported to be as low as 10 GPa approximately [18]. Therefore, we focused on developing an optimal structure with minimal defects to increase the strength of MWCNTs.

To determine such a structure, both computational and experimental studies have been performed. Recently, the introduction of crosslinks between the adjacent walls of highly crystalline MWCNTs was proven to be a successful solution. By conducting shear, compression, and pullout loading tests through molecular dynamics (MD) simulations of armchair-type MWCNTs with randomly distributed sp^3^ interwall bonding, Xia et al. [19] found that the interwall sp^3^ coupling in MWCNTs enhanced the load transfer among the walls, facilitating their complete mechanical participation; this prevented the telescoping problem during tensile testing and improved the MWCNT strength. Alternately, Peng et al. [15] reported that the fracture strength value of MWCNTs treated with controlled electron irradiation could reach as high as 80% of the value expected in defect-free SWCNTs; this is considered to be the result of crosslinking between the walls. They further confirmed through molecular mechanics approaches that the interwall load transfer improves on increasing Frenkel-pair-type crosslinks, and only a small percentage of these crosslinks is necessary to achieve optimal load transfer. In contrast with their experiment [15], the MD results obtained by Byrne et al. [20] showed that defective MWCNTs with sp^3^ interwall bonding exhibited strength values exceeding those of SWCNTs (of the same size) with crack-like defects, and concluded that composites with suitably designed MWCNTs would perform better than most SWCNT-based composites. Furthermore, Shirasu et al. [18] performed theoretical calculations and experiments to prove that the nominal tensile strength is the key factor in determining the mechanical properties when designing CNT-reinforced composites, rather than the effective tensile strength. The effective tensile strength is given by the total force divided by the area that bears the load, while the nominal tensile strength is given by the total force divided by the total area, including the hollow core. It is also highlighted in the study [18] that an effective method to improve the nominal tensile strength is to introduce crosslinks between the MWCNT walls.

Based on the aforementioned previous works, it can be assumed that an optimal MWCNT structure with an ideal crosslink density between the adjacent walls can facilitate in attaining the highest possible nominal tensile strength and Young’s modulus. It is also believed that structural optimization will have a positive influence on future experimental studies with respect to the modification of the structures of MWCNTs that are used as additives in composites. Therefore, in our study, we demonstrated the effectiveness of machine learning-assisted high-throughput MD simulations (HTMD) as a tool to overcome the difficulties of modeling and computational limitations imposed by commercial software, and understand the mechanical properties of materials with comprehensive structures. Additionally, we explore the relationship between the structural parameters/properties and mechanical properties of CNTs. We show that by combining the MD method and machine learning algorithms, it is possible to predict an optimized structure with specific structural parameters/properties that can facilitate attaining ideal mechanical properties. We believe that it would also provide some insight into the manufacturing of high-strength CNTs, especially in the synthesis of cross-linked MWCNTs through the irradiation process. Relative to the widely used interwall sp3 bonding, Frenkel-pair-type crosslinks have the advantage of enabling the simultaneous introduction of crosslinks and defects, while maintaining the bond order of carbon atoms; therefore, we preferred developing MWCNT models using Frenkel-pair crosslinks. We also focused on the nominal values of the mechanical properties of MWCNTs as they affect the quality of CNT composites.

## 2. Molecular Dynamics Models and Computational Methods

To analyze the fracture process of CNTs and investigate the influence of their structural parameters/properties on their mechanical properties, uniaxial tensile loading tests were conducted based on the MD method using the March 2018 released version open source-software, Large-Scale Atomic/Molecular Massively Parallel Simulator (LAMMPS) [21], that is developed by the Sandia National Laboratories in the United States. The adaptive intermolecular reactive empirical bond order (AIREBO) model [22] was used for the MD simulation. As it is the second-generation extension of the reactive empirical bond order potential function, the AIREBO potential function additionally considers the 12–6 Lennard-Jones potential to describe the interaction between nanotube walls, resulting from the long-range van der Waals force, thus making it suitable for calculating the potential energy of covalent bonds and the interatomic force in CNTs [23,24,25]. To obtain an optimal structure, various combinations of diameter, number of walls, chirality, and crosslink density for models were studied. To process such calculations with a high workload efficiently, the entire calculation process—from model-building and crosslink introduction to MD simulation and result determination—was controlled in the HTMD environment through Python programs and shell script algorithms. To incorporate a wide range of models, we studied SWCNTs, 2-walled CNTs (2WCNTs), 3-walled CNTs (3WCNTs), 4-walled CNTs (4WCNTs), and 5-walled CNTs (5WCNTs), with fixed lengths of 426.0 Å and 425.5 Å for zigzag- and armchair-type CNTs, respectively. To make a valid comparison, the boundary of the diameters of the zigzag- and armchair-type CNTs were assigned equal values. Corresponding to the diameters of the (21,0) zigzag-type CNTs and (12,12) armchair-type CNTs (16.4 Å and 16.3 Å, respectively) and the diameters of the (95,0) zigzag-type CNTs and (55,55) armchair-type CNTs (74.4 Å and 74.6 Å, respectively), the inner diameters of all the CNT models were set in the range of 16.3–74.6 Å. All the MWCNTs were introduced via Frenkel-pair crosslinks by controlling the density—the crosslink density is the crosslink number between two adjacent walls divided by the total number of atoms in the two walls. In the results reported by E. M. Byrne et al. [20], the 2WCNT models exhibited a “clean-break”-type fracture pattern when the crosslink density between the walls reached 2.5%. Based on this, in the present study, we set the crosslink density of our models in the range of 0–3%. In the Python-based HTMD platform, the CNT models were first generated with the desired diameter, number of walls, chirality, and crosslink density; subsequently, the models were populated with randomly distributed crosslinks. Consequently, the advantage of this method is that the time taken to complete the modeling process depends on the number of atoms in the model.

**Figure 1:**
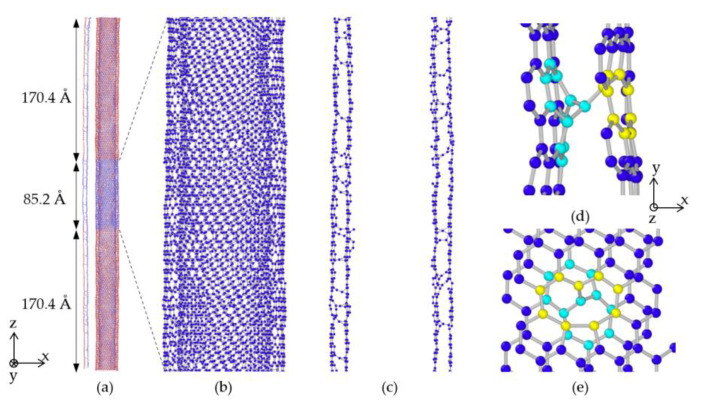
Schematic diagram of the computational model. (**a**) Entire 2WCNT with Frenkel-pair crosslinks and distortions on the wall with a half front slice on the left side, where the two red parts (on the outermost wall) indicate the regions of load application during the tensile test and are named fixed parts. The blue color (including all inner walls) represents the parts where atoms can move freely, and are thus named mobile parts; (**b**) Magnified view of the middle mobile part of 2WCNT; (**c**) Front slice of (**b**) showing randomly distributed crosslinks; (**d**,**e**) Structure of Frenkel-pair crosslink. Light blue balls represent carbon atoms on the inner walls, and yellow balls are atoms on the outer walls.

To ensure the equilibration of the internal stress and minimize the total energy for each model, an isothermal-isobaric (NPT) ensemble was coupled to a Nose–Hoover thermostat, and the relaxation process was conducted under the following conditions: 300 K temperature, 0 applied load, and 0.5 fs time step. During the equilibrium period, the minimum and maximum AIREBO potential cutoff distances were set to 1.7 Å and 1.8 Å, respectively, to facilitate better bonding of the crosslinks. To avoid the influence of thermal fluctuations on the result, the temperature was reduced to 1 K after the optimized model structure was obtained. Based on the experimental settings of the previous studies [17,26], the uniaxial tensile load was applied to the atoms of the two fixed parts of the outermost wall in the canonical ensembles (NVT) along the z-axis, with the engineering strain rate controlled at 6 × 10^9^ s^−1^ and time step at 0.5 fs. Please note that the fixed part also experienced elongation along the tube axis as the load increased, while all the atoms in the mobile part including the middle portion of the outermost wall and all inner walls could move freely, as shown in Figure 1. To avoid non-physical increases in stress values during tensile loading, the AIREBO potential cutoff distance was modified to 2.0 Å [27,28,29]. The resultant tensile strength of the AIREBO potential–based tensile test verification simulation of the zigzag-type CNTs obtained in this study was 120 GPa. This was slightly higher than the experimental result, and consistent with those of the quantum calculation obtained by B. Peng et al. [15], which were approximately 100 GPa and 120 GPa. Moreover, the strain-stress relation for single-walled fracture of MWCNTs was in good agreement with their experiment sample 1, 2 and 3. Because the distribution pattern of the crosslinks may influence the result and the interwall crosslink density has at most 3% margin of error after equilibration, at least three calculations were performed for each MWCNT model, and the average values were considered to be the final results.

For each model, the values of strain, stress, nominal tensile strength, and nominal Young’s modulus were obtained. The deformation along the z-axis of the model was divided by its original length to obtain the strain, and the stress was determined by dividing the stress tensor by the volume of the carbon atoms, where the stress tensor was obtained using LAMMPS. The nominal tensile strength was calculated by dividing the product of the fracture stress and effective area by the entire cross-sectional area. The effective area comprises the tension loading cross-sectional areas, including the wall thickness of the model, as shown in Equation (1), and the nominal area is the full cross-sectional area of the outermost wall including the wall thickness, as shown in Equation (2):

(1) $$A_{\mathrm{eff}}=\pi \left[{\left(r_{\mathrm{out}}+0.5t\right)}^{2}-{\left(r_{\mathrm{in}}-0.5t\right)}^{2}\right]$$

(2) $$A_{\mathrm{nom}}=\pi {\left(r_{\mathrm{out}}+0.5t\right)}^{2}$$

where $A_{\mathrm{eff}}$ and $A_{\mathrm{nom}}$ are the effective area and the nominal area, respectively; $r_{\mathrm{in}}$ and $r_{\mathrm{out}}$ are the radii of the innermost wall and outermost wall, respectively; and t is the wall thickness. The nominal Young’s modulus was calculated by dividing the nominal stress by the strain during elastic stretching. When calculating the area, the thickness of the individual wall was considered to be 3.4 Å.

**Figure 2:**
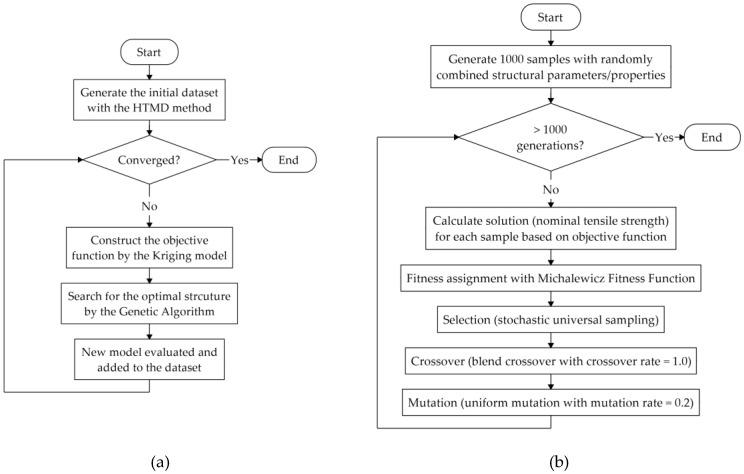
Flowchart of the optimization procedure. (**a**) Bayesian optimization; (**b**) genetic algorithm.

As suggested in previous studies, we focused on structural optimization, particularly with respect to the nominal tensile strength. To optimize the structural parameters/properties for CNTs, a machine learning algorithm, namely the Bayesian optimization method [30,31], was used. A flowchart of this algorithm is shown in Figure 2a. Three basic steps were included in the Bayesian optimization adopted in this study: The first step involves the construction of the objective function using the Kriging model [32,33] and the prediction of the optimal nominal tensile strength value based on the acquisition function by evaluating the expected improvement [33]. In the second step, a genetic algorithm (GA) [34,35,36] was introduced to determine the structural parameters/properties for the CNTs, which can achieve the predicted mechanical property, as shown in Figure 2b. Specifically, 1000 samples structured with randomly selected structural parameters/properties, including diameter, number of walls, chirality, and crosslink density, were generated as the population for the 1st generation; subsequently, the solution (nominal tensile strength) of each sample was calculated based on the objective function predicted by the Kriging model from the former step. To determine the fitness parameter in the GA, the Michalewicz fitness function [37] was used, and solutions in the population were ranked using the Fonseca-Fleming method [38] in objective function space. The fitness was then assigned to each solution based on its rank. By using the stochastic universal sampling method [39], better solutions were selected as parents to produce the next generation. During this process, blend crossover [40] was performed with a crossover rate of 1.0, and uniform mutation [36] with a mutation rate of 0.2 was used. The procedure was repeated for 1000 generations, and an optimal structure with a set of structural parameters/properties was returned as the result of the GA. In the third step, the optimal structure was evaluated by the MD simulation to obtain the real nominal tensile strength value. This value was then added along with those of the structural parameters/properties to the dataset as a new model. By repeating steps one to three, the structural parameters/properties were gradually optimized as the predicted parameters for new model approached stability. With respect to the calculation cost, 97 sets of models were evaluated using our Bayesian optimization method, while 56 models with randomly selected structural parameters/properties were considered to be the initial database.

To visualize the optimized structure parameters/properties, a self-organizing map (SOM) was used. As an effective data mining approach for data-driven materials study, an SOM can reduce high-dimensional data to two-dimensional maps based on the neural network model constructed using unsupervised competitive learning algorithms, while preserving the topology of the data [41,42]. In this study, a commercial software developed by the company Viscovery in Austraia, namely Viscovery^®^ SOMine 5.2.2 Expert Edition [43], was employed to produce SOMs. In addition to the general SOM algorithm, a Kohonen’s batch map based on an advanced unsupervised neural network [44] was deployed inside the software. For parameters in SOM, the number of nodes and topology were set to 1000 and hexagonal, respectively.

## 3. Results

**Figure 3:**
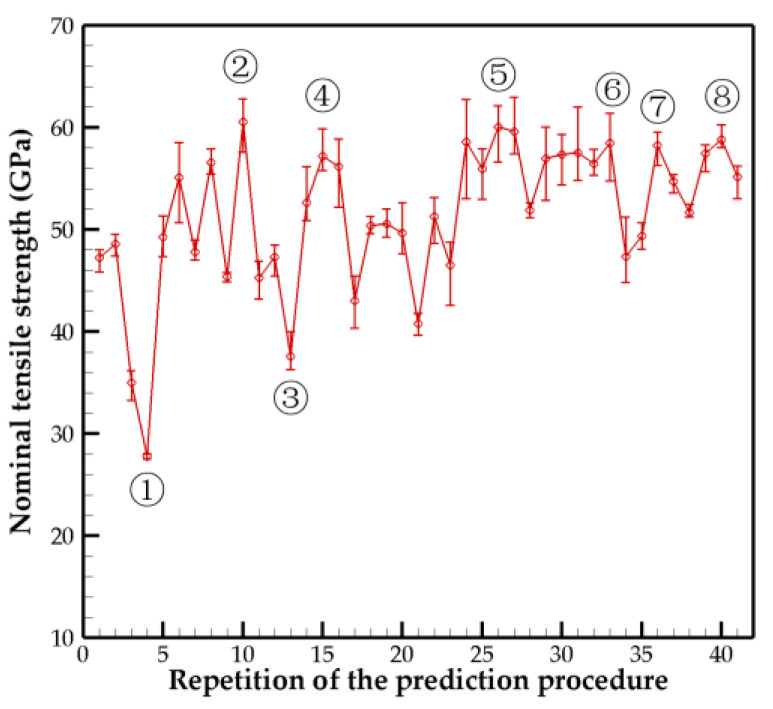
Strength results of the machine learning–predicted structure with respect to the repetition of the prediction procedure. Error bar shows the maximum and minimum values obtained from the three repetitions of calculations.

In all the results, the margin of error of the tensile strength was less than 5%, which was caused by the error margins of both the crosslink density and the crosslink distribution difference. Based on our calculations, the structure optimization was performed 41 times. The tensile strength results for each predicted model are shown in Figure 3, and their representative values are listed in Table 1. It can be observed that there are fluctuations in the initial prediction results; with the repetition of the prediction procedure, the tensile strength of the newly predicted structure tends to stabilize. However, the values in Table 1 indicate that although the chirality, number of walls, and diameter become stable, the predictions of the crosslink density for each adjacent wall continue to fluctuate. This is due to the influence of crosslink distributions and the presence of margin errors. Therefore, we combined the prediction models corresponding to the 10 highest tensile strength values as the final result. The armchair-type 5WCNT was concluded to be the optimal structure with high mechanical performance, where the outer diameter was 43.39 Å; the crosslink densities between the adjacent walls from the inner to outer tubes were 1.38 ± 1.16%, 1.13 ± 0.69%, 1.54 ± 0.57%, and 1.36 ± 0.35%; and the nominal tensile strength and nominal Young’s modulus values were approximately 58–64 GPa and 677–698 GPa, respectively. The effective tensile strength and Young’s modulus values based on the effective cross-sectional area (Equation (1)) were slightly higher than nominal values and range from approximately 65–71 GPa and 730–754 GPa, respectively.

**Figure 4:**
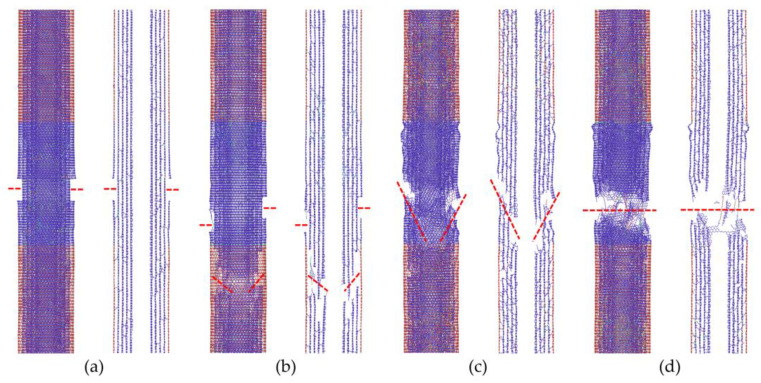
Fracture patterns of the zigzag-type 5WCNTs assigned with different crosslink densities. (**a**) “Sword-in-sheath” fracture on the 0.5% crosslink model (in every wall) with nominal strength of 47 GPa; (**b**) “Sword-and-sheath” fracture on the 0.75% crosslink model with nominal strength of 50 GPa; (**c**,**d**) “Near-clean-break” and “clean-break” fracture on the 1.5% crosslink model with nominal strength of 53 GPa and 52 GPa, respectively.

We further confirmed the fracture pattern transition with the increase in crosslink densities, as shown in Figure 4. In the case of the low crosslink density shown in Figure 4a, the load being transferred from the outermost wall onto the inner walls is limited, which leads to the fracture of only the outer tube, i.e., the “sword-in-sheath” fracture mode. The intermediate crosslink density can help improve the load transfer between the walls; however, the failure of the inner tube often occurs at a position away from the fracture plane of the outer wall, as shown in Figure 4b; this is known as the “sword-and-sheath” fracture mode. Conversely, for high crosslink density in MWCNTs, a “near-clean-break” fracture mode, which is depicted in Figure 4c, is often observed in the results, while the “clean-break” fracture mode appears in some cases as a special pattern of the “near-clean-break” fracture mode, as presented in Figure 4d. This result can be attributed to the different distribution patterns of the crosslinks between the walls.

**Figure 5:**
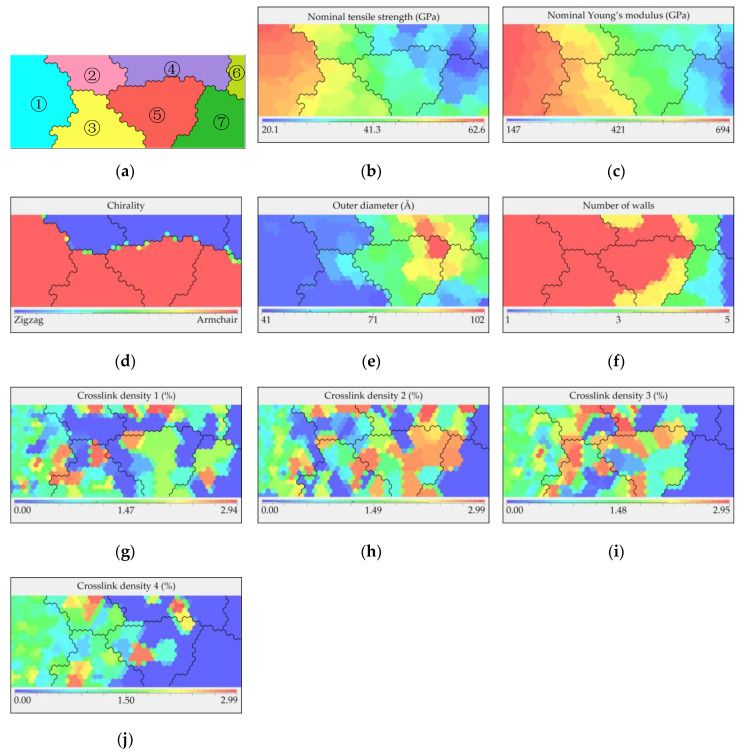
SOM results including the cluster map and heat map. (**a**) Cluster map with 7 clusters; (**b**) Heat map of nominal tensile strength: values range from 20.1 GPa to 62.6 GPa; (**c**) Heat map of nominal Young’s modulus: values range from 147 GPa to 694 GPa; (**d**–**f**) Heat map of chirality, diameter, and number of walls, respectively; (**g**–**j**) Heat maps of crosslink density 1–4, where crosslink density 1 is the density corresponding to the innermost wall, and crosslink density 4 is the density corresponding to the outermost wall.

To assist in visualization, an SOM was used to reduce the high-dimensional results from the database into two-dimensional figures, as displayed in Figure 5. The priority parameter of the SOM was set to the nominal tensile strength so that the relationship among the structural parameters/properties could be revealed clearly. All other properties, including the nominal Young’s modulus, were mapped according to the tensile strength result of each model. The SOM consists of two parts: the cluster map and heat maps, where the two dimensions of these maps do not represent any variables. Figure 5a shows the cluster map with seven clusters that are automatically divided, where each cluster represents the CNTs with similar nominal tensile strengths. Figure 5b–j are heat maps corresponding to the specific parameters, with different colors distinguishing the high and low values. As shown in Figure 5b,c cluster 1 appears to exhibit better performance based on the nominal values of the mechanical properties. By combining Figure 5d–f, it can be observed that most of the models within cluster 1 are armchair-type CNTs with the smallest outer diameters and many walls. Moreover, when comparing Figure 5g–j, the crosslink density in cluster 1 varies from dispersion to gradual concentration from “crosslink density 1” to “crosslink density 4,” where “crosslink density 1” is the density between the innermost layer and its adjacent layer, while “crosslink density 4” is the density between the outermost wall and its adjacent wall. Based on the observation, among all the 5WCNT models, “crosslink density 4” is significant to the overall strength of the tubes. Thus, it can be concluded that the influence of the crosslink density between the layers on the overall strength of the tube decreases from high to low from the outside to the inside, and when “crosslink density 4” reaches a value of approximately 1–1.8%, the value of the other crosslink densities become less important.

## 4. Discussion

**Figure 6:**
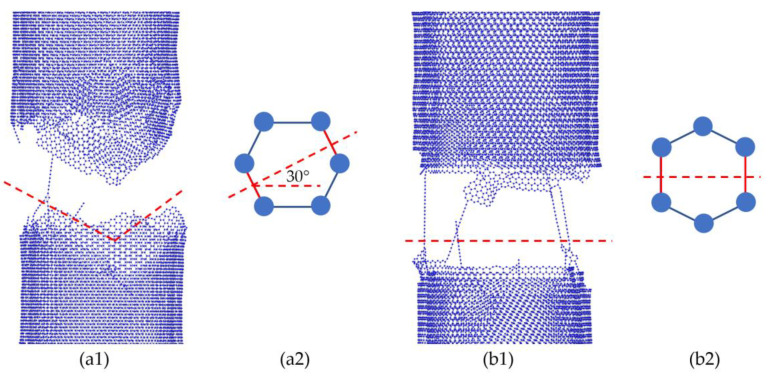
Fracture behavior difference between zigzag-type and armchair-type 2WCNTs. (**a1**) Approximately 30° fracture for armchair-type CNT; (**b1**) Nearly linear fracture for zigzag-type tube; (**a2**,**b2**) Atom arrangement of armchair-type CNT and zigzag-type CNT, respectively.

In our study, the relationship between structural parameters/properties and mechanical properties were investigated. It was evident that achieving a high nominal tensile strength requires armchair-type MWCNTs with small diameter, large number of walls, and ideal crosslink densities between the adjacent walls. Such a result can be explained as follows: as shown in Figure 6, the different atom arrangements in armchair-type CNTs and zigzag-type CNTs (Figure 6(a2,b2), respectively) lead to the load bearing difference, causing a fracture of approximately 30° in armchair-type CNTs (Figure 6(a1)) and a nearly linear fracture for zigzag-type CNTs (Figure 6(b1)). As a result, armchair-type CNTs have a higher tensile strength and better elongation properties than those of zigzag-type CNTs. Similar results were also obtained by other researchers [23,45,46].

For the calculation of nominal mechanical property values, the hollow core of CNTs is considered to have a significant impact. During the tensile test, the hollow core did not bear any load; nevertheless, it was incorporated in the stress calculation process. To minimize the influence of the hollow core section, a small-diameter tube was required. The reason for the preference of many walls is that with an optimized crosslink density, more walls can share the load applied on the outermost wall. Even if there are no crosslinks between the walls, the van der Waals force in the adjacent walls can transfer a limited amount of load onto the inner walls.

Furthermore, our investigation on the influence of the crosslink density between the layers on the overall strength of the tube revealed that it reduces from the outer tube to the inner tube, and can provide practical guidance for high-performance-CNT synthesis. Since the commonly used crosslink-introduction method of irradiation is likely to create more crosslinks in the outer tube than inner tube [15,47], we suggest that it is necessary to determine the amount of irradiation dose that will produce sufficient crosslinks on the outermost wall, and not to overly focus on whether the crosslink density is at the right level for each inner wall. However, our result was limited to determining the optimal structure for 5WCNTs, and although we demonstrated that many walls are preferred, it is uncertain whether the crosslink density value for the outermost wall of 1.36 ± 0.35% is most suitable for MWCNTs with larger than five walls.

## 5. Conclusions

We presented a novel approach to predict the optimal structure of high-mechanical performance CNTs through machine learning-based simulations in the HTMD environment. Based on the results obtained for the structural parameters/properties of diameter, number of walls, chirality, and crosslink density, we concluded that to achieve a high nominal tensile strength, armchair-type MWCNTs with the smallest diameter, large number of walls, and a suitable crosslink density between the adjacent walls are preferred. Based on our calculations, the armchair-type 5WCNT—with the outer diameter of 43.39 Å, the crosslink density between adjacent walls (from inner tube to outer tube) of 1.38 ± 1.16%, 1.13 ± 0.69%, 1.54 ± 0.57%, 1.36 ± 0.35%—exhibits the best mechanical properties. The nominal tensile strength, nominal Young’s modulus, effective tensile strength, and effective Young’s modulus were approximately 58–64 GPa, 677–698 GPa, 65–71 GPa, and 730–754 GPa, respectively. We further discussed the relationship between fracture pattern and mechanical properties of CNTs, and it was observed that the tubes with “near-clean-break” fracture mode and “clean-break” fracture mode tend to exhibit a high tensile strength. By analyzing the data through SOM visualization, we explained the reason behind the specific structural parameters/properties facilitating a high mechanical performance, and showed that the influence of crosslink density on the mechanical properties tends to gradually decrease from the outer walls to the inner walls. The proposed method and obtained results presented a valuable approach to understand the mechanical properties of CNTs and provided guidance on the tailoring of CNT structures to improve the quality of composites.

## Figures and Tables

**Table 1:** Detailed information for the representative values in Figure 4. “Crosslink density 1” through “Crosslink density 4” represent the crosslink densities between each adjacent wall from the inner tube to the outer tube. The average value is given, and the range is indicated in parentheses.

Model	Outer Diameter (Å)	Number of Walls	Chirality	Crosslink Density 1 (%)	Crosslink Density 2 (%)	Crosslink Density 3 (%)	Crosslink Density 4 (%)	Nominal Tensile Strength (GPa)
①	101.70	5	Armchair	0.01 (0.01–0.01)	2.32 (2.31–2.32)	2.36 (2.35–2.37)	2.26 (2.25–2.28)	27.79 (27.53–28.04)
②	44.75	5	Armchair	0.91 (0.89–0.92)	0.75 (0.74–0.77)	0.97 (0.95–0.97)	1.38 (1.35–1.41)	60.53 (57.60–62.81)
③	44.62	5	Zigzag	0.02 (0.02–0.02)	0.83 (0.81–0.85)	0.05 (0.04–0.05)	1.70 (1.69–1.71)	37.58 (36.28–40.02)
④	43.39	5	Armchair	2.85 (2.81–2.90)	1.19 (1.18–1.21)	1.47 (1.45–1.48)	1.83 (1.81–1.84)	57.19 (55.80–59.88)
⑤	43.39	5	Armchair	0.22 (0.20–0.23)	0.44 (0.44–0.45)	2.11 (2.10–2.12)	1.20 (1.20–1.21)	60.03 (56.62–62.12)
⑥	43.39	5	Armchair	0.05 (0.04–0.05)	2.03 (2.01–2.05)	1.32 (1.30–1.35)	1.32 (1.31–1.33)	58.50 (54.77–61.42)
⑦	43.39	5	Armchair	1.98 (1.97–2.00)	0.84 (0.82–0.86)	0.59 (0.57–0.60)	1.01 (1.00–1.03)	58.26 (56.25–59.52)
⑧	43.39	5	Armchair	0.90 (0.89–0.92)	1.39 (1.36–1.41)	1.40 (1.40–1.41)	1.25 (1.23–1.28)	58.83 (58.02–60.26)
